# Mechanical Properties and Mechanism of Geopolymer Cementitious Materials Synergistically Prepared Using Red Mud and Yellow River Sand

**DOI:** 10.3390/ma17153810

**Published:** 2024-08-02

**Authors:** Weizhun Jin, Yiming Chen, Yajun Lv, Linhua Jiang, Weifeng Bai, Kangjie Zhang, Caihong Song, Xianlei Zhang

**Affiliations:** 1 College of Civil Engineering, Henan University of Engineering, Zhengzhou 451191, China; j_weizhun@163.com; 2 Henan Provincial Technical Center for Ecology and Environment, Zhengzhou 450004, China; 3 School of Architecture, North China University of Water Resources and Electric Power, Zhengzhou 450046, China; chenyiming0528@163.com (Y.C.); k1529647428@163.com (K.Z.); s2105191006@163.com (C.S.); 4 Henan Engineering Technology Research Center of Eco-Environmental Damage Identification and Restoration, Zhengzhou 450004, China; 5 College of Civil and Transportation Engineering, Hohai University, Nanjing 210024, China; hhulhjiang@163.com; 6 School of Water Conservancy, North China University of Water Resources and Electric Power, Zhengzhou 450046, China; baiweifeng@ncwu.edu.cn

**Keywords:** red mud, Yellow River sand, geopolymer cementitious materials, compressive strength, low carbon emission

## Abstract

In order to reduce the negative impact on the environment caused by the massive accumulation of red mud (RM) and Yellow River sand (YRS), new alkali-excited granulated blast-furnace slag (GGBS)/RM/YRS (AGRY) geopolymer cementitious materials were prepared by combining RM and YRS with GGBS in different ratios and using sodium silicate as the alkali exciter. The effects of YRS dosage and different curing conditions on the mechanical properties, hydration products, and pore structure of cementitious materials were investigated and analyzed in terms of cost and carbon emissions. The results showed that when the dosage of YRS was 40%, the compressive strength of the prepared AGRY cementitious material could reach 48.8 MPa at 28 d under standard curing, which showed mechanical properties comparable to those of the cementitious material without YRS. The cementitious material has a more compact internal structure, and the combination of RM and YRS promotes the chemical reaction of Al and Si elements and generates the (N, C)-A-S-H gel products, which are the key to the strength enhancement of the cementitious material. In addition, the prepared cementitious material is only 90% of the cement cost for the same strength and has low carbon emission accounting for only 43% of the cement carbon emission. This study not only provides a new way for the resource utilization of RM and YRS, but also contributes an excellent new environmentally friendly material for the construction industry to achieve the goal of low carbon development.

## 1. Introduction

As the world continues to industrialize, carbon emissions are having a huge impact on global climate [1,2]. In response to the adverse impacts of climate change on the human environment, several countries around the world have proposed carbon-neutral targets. The construction sector, as a key to industrialization, accounts for more than 40% of global carbon emissions [3]. As a result, many scholars have begun to focus on conducting research on carbon reduction in the construction industry [4,5,6]. The production process of cement, the core of building materials, is one of the main sources of industrial carbon emissions. The main source of carbon generated by cement comes from the high temperature calcination during its production (decomposition of CaCO_3_ when heated to 1100 °C) [7,8]. Meanwhile, the energy consumption in the process of calcination of limestone to produce lime at a high temperature also emits a large amount of CO_2_ [9]. Carbon emissions from the cement industry are likely to continue to increase in the future and contribute to a number of environmental problems such as global warming [10,11,12]. Therefore, finding new environmentally friendly cement alternative materials to reduce carbon emissions has become an urgent problem to be solved.

Red mud (RM) is an industrial waste produced during alumina production, with a global annual production of about 12.01 billion tons per year [13]. However, the utilization rate of RM is only about 1% [14]. For every ton of alumina produced, 1–2 tons of RM are generated [15]. As an industrial by-product of high alkalinity [16], RM’s fineness, toxicity, and radioactivity pose a great threat to the living environment of humans [17,18,19]. Most of the current RM treatment methods are based on damming and storage, which occupies a large amount of arable land, poses safety hazards, and pollutes the environment. Meanwhile, many researchers have studied the recycling of RM. It can be used to make ceramic products [20], gelling materials [15,21,22,23,24], retarders [21], and pH buffers [25], among others. To date, there has been an increasing amount of research related to RM waste utilization. However, it can be practically applied in the field of building materials in only a few countries. Furthermore, RM can replace less than 5% of the volume of a specific building material, such as cement, and the rest is stored as waste [26]. China is the largest producer of primary aluminum in the world (56.3% of the world’s supply), and Henan Province is a large aluminum-producing province with the second largest aluminum production in the country [27]. With an annual increase of more than 15 million tons of RM in urgent need of treatment [28], there is an urgent need to treat and make effective use of it to alleviate the great pressure of RM on environmental protection.

The Yellow River is considered to have the highest sediment load in the world, delivering an average of 1.1 billion tons of sediment to the ocean each year [29], which accounts for about 6% of the total global river sediment [30]. In addition to the sediment flowing into the ocean, 26% of the sediment is silted up in the riverbed [31], which causes the riverbed to rise and leads to the Yellow River becoming a dangerous aboveground overhanging river [32]. Therefore, how to deal with its sediments has become one of the key issues in protecting the Yellow River. In recent years, some researchers have studied the utilization of Yellow River sand (YRS) [33]. Li et al. [34] prepared a cementitious material with a strength of 20.3 MPa using Yellow River silt as raw material with fly ash (FA). Wang et al. [35] used Yellow River silt as a raw material combined with blast furnace slag to prepare a cementitious material with strength up to 12.3 MPa. He et al. [36] studied the combination of Yellow River silt and RM for the preparation of sintered bricks with a strength of up to 39 MPa, as well as the characteristics and mechanism of the preparation process. However, most of current research is to prepare building materials from YRS through secondary sintering, which consumes a large amount of energy and does not reduce carbon emissions during its preparation. Therefore, it is of higher relevance to adopt a low-carbon approach to YRS and RM.

Geopolymer (also known as alkali-excited cementitious materials) is characterized by fast setting speed [37,38], high temperature resistance [39,40,41], and corrosion resistance [42,43,44]. Geopolymer can be prepared without the need for calcination, offering great potential to reduce carbon emissions by replacing cement. In recent years, there has been an increasing amount of research related to alkali-excited cementitious materials [45,46,47,48]. Currently, the matrix materials used in alkali excitation-related studies are mainly based on granulated blast-furnace slag (GGBS), FA, metakaolin, silica fume, and rice husk ash [49,50,51,52]. Meanwhile, the geopolymer cementitious materials have been used in various construction applications and some methods for improving their mechanical properties have been investigated [53,54,55,56]. Jonathan et al. [57] studied the mechanical properties of geopolymer concrete using a pumice-derived sodium silicate solution. The results revealed that the 56-day strength of the geopolymer concrete was 22.4 MPa, which was approximately a 19% increase compared with the 28-day strength. Ahmed et al. [58] studied recycled brick waste powder (RBWP) in engineered geopolymer composites and the results demonstrated that when RBWP completely replaced FA, the compressive strength, tensile strength, and tensile strain capacity increased by 25%, 29%, and 172%, respectively. However, some of the curing conditions of the geopolymer cementitious materials need to be carried out at high temperatures [59] and its high manufacturing cost is also a hindrance to its application in practical engineering [60,61,62]. Therefore, the preparation of low-carbon alkali-excited cementitious materials requires more in-depth research. Further, the silicon to aluminum ratio (Si: Al) in the geopolymer is decisive for the degree of polymerization of the material [63]. Si and Al are mainly derived from alkali exciters and raw materials, and the choice of these directly affects the formation of gelling products in geopolymer concrete, which is crucial for improving the strength of alkali-excited concrete. RM and YRS contain elements such as Si and Al that can provide a large number of elements needed for alkali-excited cementitious materials, so it is expected that the combination of RM and YRS can produce new building materials with considerable strength by adopting suitable techniques. There are relatively few studies on the preparation of RM/YRS cementitious materials adopting low-carbon and environmentally friendly alkali-excited techniques.

Based on the current research status, it can be concluded that RM needs to be treated on a large scale in a more environmentally friendly way, and the large-scale utilization of YRS is crucial to the dredging of the Yellow River. Furthermore, the large-scale utilization of RM and YRS in the form of alkali-excited geopolymers can not only effectively solve the ecological problems caused by RM and YRS but can also replace cement as a new building material to reduce carbon emission. However, few studies have been conducted on the preparation of geopolymer by means of alkali excitation using RM and YRS, and lack of studies on the effect of different curing methods on the properties of geopolymers prepared by RM and YRS makes it urgent to carry out relevant research in this area. Therefore, an attempt was made in this study to prepare alkali-excited GGBS/RM/YRS (AGRY) cementitious materials. The effects on workability, mechanical properties, hydration products, and pore distribution were investigated by varying the raw material mixing ratios and curing methods of the AGRY cementitious materials, and the carbon emission and cost analysis were carried out. Meanwhile, the formation mechanism of AGRY gel products was investigated and the feasibility of using the synergistic effect of RM and YRS to prepare geopolymer cementitious materials and realize their applications was analyzed.

## 2. Materials and Methods

### 2.1. Materials

**Figure 1:**
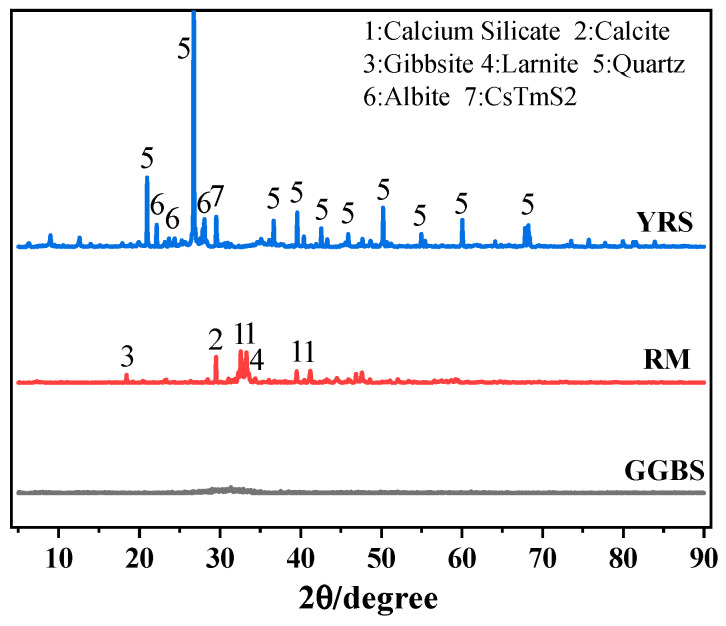
XRD patterns of GGBS, RM, and YRS.

RM was provided by Aluminum Corporation of China Co., Ltd. (Hong Kong, China) The sintered RM was dried in an oven at 105 °C for 24 h and passed through a sieve of 0.16 mm after dry grinding for 30 min using a ball mill. YRS was obtained from the coastal area of Henan Province, downstream of the Yellow River. The YRS was dried in an oven at 105 °C for 24 h and sieved with a sieve of 0.075 mm. GGBS was provided by Gongyi Longze Water Purification Materials Co., Ltd., Gongyi, China. The XRD patterns of RM, YRS, and GGBS are shown in Figure 1. From Figure 1, it can be seen that the main mineral components in RM are calcium silicate, calcite, alumina trihydrate, and plagioclase. The mineral components in YRS are mainly quartz and sodium feldspar. GGBS is amorphous, with no obvious crystalline phase and a very broad hump at 30~40°. Meanwhile, the chemical compositions of RM, GGBS, and YRS were analyzed by X-ray fluorescence (XRF) and the results are shown in Table 1.

The alkali exciter adopts instant sodium silicate with modulus 2.3, provided by Henan Platinum Run Casting Materials Co., Ltd., Gongyi, China. Sodium lignosulfonate was selected as the water reducing agent in the experiment.

### 2.2. Specimen Preparation

To design the mix ratio, the ratio of GGBS to RM/YRS was fixed at 3:7, the mass fraction of GGBS was kept constant, and the mass ratio of RM to YRS was varied with mass ratios of 80:20, 60:40, 40:60, and 20:80, respectively. The mix proportions are listed in Table 2. Considering the economy, the modulus of the alkali exciter was chosen to be 2.3 [64]. In this study, the water to cementitious materials (W/(RM + GGBS + YRS)) ratio was 0.4, and the amount of water-reducing agent was 0.5% of the mass of cementitious materials.

**Figure 2:**
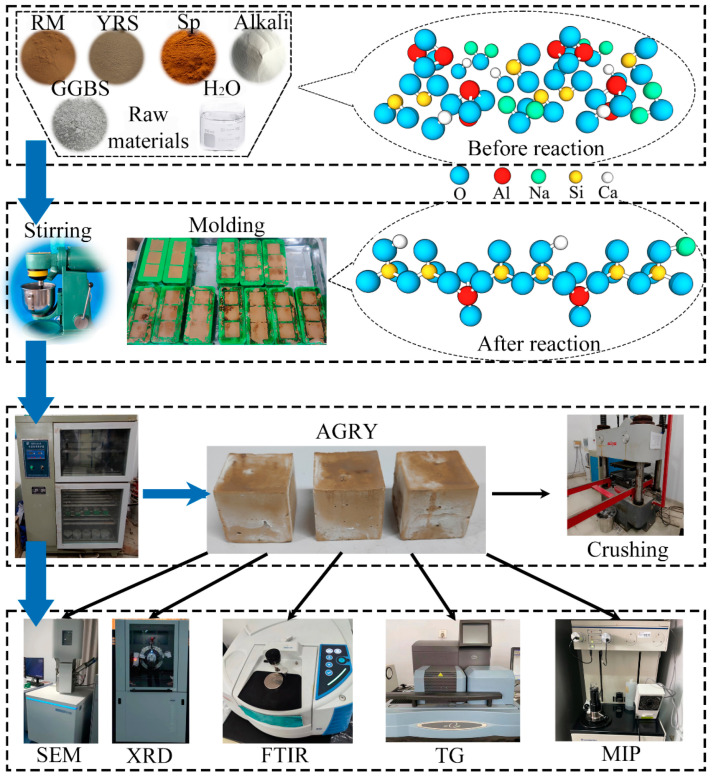
Preparation process of AGRY cementitious materials.

The preparation process of AGRY cementitious materials is shown in Figure 2. First, the alkali exciter, water reducer, and water were mixed and stirred at 400 rpm for 5 min until the alkali exciter and water-reducing agent dissolved to prepare an alkali excitation solution. Then, the raw materials of RM, GGBS, and YRS were mixed and stirred for 2 min to obtain the evenly dispersed mixture, and then the alkali excitation solution was poured into the evenly dispersed mixture and stirred for 3 min. Next, the fresh mortar mixture was formed in a mold of 40 mm × 40 mm × 40 mm. The AGRY cementitious materials were obtained by removing the molds after 24 h of curing at room temperature. Finally, the AGRY cementitious materials were cured using two methods: (I) the natural curing condition of 25 ± 5 °C and RH of 30~60%; (II) the standard curing condition of 20 ± 2 °C and RH of 95%. The AGRY cementitious materials cured for 3 d, 7 d, and 28 d were tested for compressive strength, and the fragments obtained during the compressive strength test were analyzed for hydration products and microstructure.

### 2.3. Workability and Mechanical Strength of AGRY Cementitious Materials

The setting time of the AGRY paste was tested according to Chinese standard GB/T 1346-2011 [65]. During the test, the AGRY paste was poured into a truncated cone mold. The initial setting time was the time recorded when the paste was poured into the mold until the needle drops freely 4 mm from the bottom. When the needle fell into the paste no more than 0.5 mm, the recorded time was the final setting time. 

The fluidity of the AGRY paste was tested according to Chinese standard GB/T 8077-2012 [66]. In the test, the AGRY paste was poured into the truncated cone mold, and the mold was lifted vertically to let the paste sag naturally, allowing the paste to flow on the glass plate for 30 s. Then, the maximum diameter of the paste in two directions perpendicular to each other was measured with a ruler, and the average value was taken as the fluidity of the AGRY paste.

The mechanical strength of the AGRY cementitious materials was tested according to Chinese standard GB/T 17671-2021 [67]. During the test, a microcomputer-controlled electro-hydraulic servo pressure tester was used to test the compressive strength of the AGRY cementitious materials. During loading, the sample was uniformly loaded at 2400 ± 200 N/s until it was destroyed. The average compressive strength of three samples was taken as the final compressive strength.

### 2.4. Hydration Products and Microstructure of AGRY Cementitious Materials

Before testing, the crushed pieces after compressive strength test were soaked in anhydrous ethanol to stop hydration and dried at 60 °C for 24 h. X-ray diffractometer (XRD) was used to test the samples at 28 d under different conservation conditions. The scanning angle range was selected from 5 to 90°, and the scanning rate was 2°/min. The wavelength, voltage, and current were 1.5418, 40 kV, and 40 mA, respectively. The chemical bonding of samples was analyzed using Fourier transform infrared (FTIR) spectroscopy. The infrared spectrometer had a wavelength range of 400 to 4000 cm^−1^ and a resolution of 1 cm^−1^. The samples were analyzed by thermogravimetric (TG) analysis using a simultaneous thermal analyzer with the temperature range set from 25 to 1000 °C. The continuous loss of weight of the samples was recorded at a heating rate of 10 °C/min. Scanning electron microscopy (SEM) was used to test the microscopic morphology of the internal fragments of the samples, and the specimens were analyzed for their compositions using an energy dispersive spectrometer (EDS). The pores of the sample fragments were tested using a high-performance mercury intrusion porosimetry (MIP) instrument. The volume of the fragments was 10 mm × 10 mm × 2 mm. The pressure ranged from 0.15 to 300 MPa.

### 2.5. Cost and Carbon Calculation of AGRY Cementitious Materials

One of the most important factors determining whether new building materials can be applied to the actual production process is the cost of production. The costs of raw materials, transportation, and material preparation need to be considered when calculating the total cost of the new material. Equation (1) represents the process of calculating the total cost of material preparation [68].

(1) $$C_{T}=\sum_{i=1}^{n}\left(C_{MP}+C_{MT}\right)\times Q_{i}+C_{EU}$$

where $C_{T}$ is the total cost; $C_{MP}$ is the cost of the substrate material; $C_{MT}$ is the cost of transportation of materials; $Q_{i}$ is the amount of each material required per 1 m^3^ of AGRY prepared; $C_{EU}$ is the cost of energy required to manufacture AGRY.

The total CO_2_ emissions from AGRY cementitious materials (*CO*_2−_*_T_*) consist of CO_2_ generated during the production phase of raw materials and CO_2_ during the transportation phase of materials. *CO*_2−_*_T_* is calculated as shown in Equation (2).

(2) $$CO_{2-T}=\sum_{i=i}^{n}\frac{Q_{i}}{Lt}\times \frac{d}{e}\times F_{iT}+\sum_{i=i}^{n}P\times t\times F_{iP}$$

where Lt is the weight of the material; d is the transportation distance; e is the efficiency of energy conversion during transportation; $F_{iT}$ is the CO_2_ emission factor during transportation; P is the power of the machine used in the preparation process of AGRY; t is the machine usage time; $F_{iP}$ is the CO_2_ emission factor in the production process.

## 3. Results and Discussion

### 3.1. Fluidity and Setting Time

**Figure 3:**
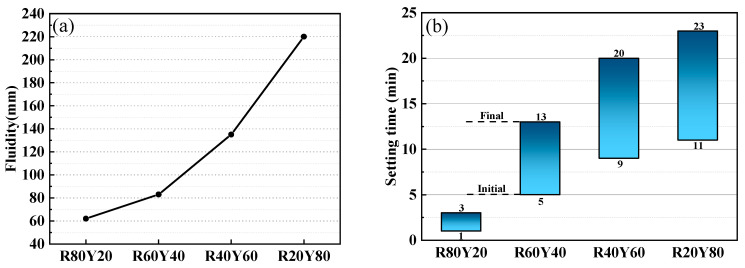
Workability of AGRY pastes: (**a**) fluidity; (**b**) setting time.

The fluidity and setting time of AGRY pastes are shown in Figure 3a,b. From Figure 3a, it can be seen that the fluidity of AGRY pastes increases with the increase of YRS dosage. When the YRS dosage is 20% of the total mass of the cementitious material (the mass ratio of YRS to RM is 20: 80), the fluidity of the paste is 61 mm. As the dosage of YRS increases to 80% (the mass ratio of YRS to RM is 80: 20), the fluidity of the paste increases to 220 mm. At this time, the fluidity of the paste can basically meet the requirements of the workability of the cementitious materials, and it also shows that the addition of YRS will improve the fluidity of the paste. The reason for this result is mainly due to the fact that RM has a smaller particle size and a larger specific surface area compared to YRS [69], so when the dosage of RM in the AGRY paste is too high, it leads to a decrease in the flowability of the paste.

From Figure 3b, it can be seen that the setting time of AGRY paste decreases with the increase of RM dosage. When the RM dosage is 20%, the initial setting time and final setting time are 11 min and 23 min, respectively. When the RM dosage is 80%, the initial and final setting times are 1 min and 3 min, respectively. This is mainly due to the accelerated alkali excitation reaction due to the high dosage of RM, resulting in accelerated setting phenomenon, especially when the dosage of RM is 80%, the setting time is 1 min, which produces the flash setting phenomenon.

### 3.2. Compressive Strength

**Figure 4:**
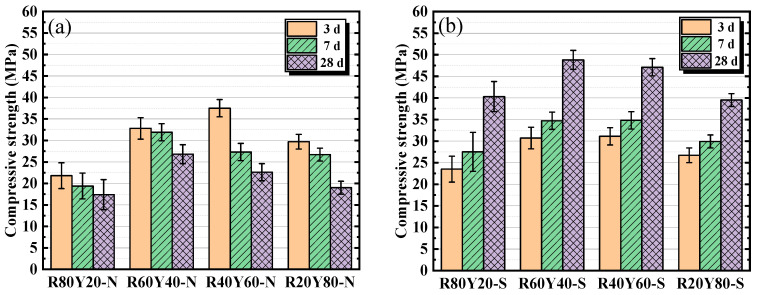
Compressive strength of AGRY cementitious materials under different curing conditions: (**a**) Natural curing; (**b**) Standard curing.

The compressive strength of AGRY cementitious materials under natural curing and standard curing at different ages is shown in Figure 4. From Figure 4a, the compressive strength decreases gradually with increasing age under natural curing. Taking R60Y40-N as an example, the compressive strength at 3, 7, and 28 d is 32.8, 31.9, and 26.8 MPa, respectively. This is mainly due to the fact that under natural curing, the relative humidity is only 30–60%, and the water inside the cementitious materials is constantly evaporating. This will result in an aqueous environment that cannot satisfy the occurrence of depolymerization reaction and slow down the process of alkali excitation reaction which leads to a difficulty in the improvement of strength. Evaporation of moisture can also result in drying cracks within the cementitious materials leading to the decrease of strength. From Figure 4b, the compressive strength gradually increases with increasing age under standard curing. Taking R60Y40-S as an example, the compressive strength at 3, 7, and 28 d is 30.7, 34.7, and 48.8 MPa, respectively. This is mainly due to the fact that under standard curing, the cementitious materials will continue to undergo alkali excitation reaction under sufficient moisture, thus generating more hydration products with cementitious ability and gradually improving the strength with the increasing age.

It can also be seen from Figure 4 that the compressive strength of AGRY cementitious materials tends to increase and then decrease when the mass ratios of RM and YRS are from 80: 20 to 20: 80. This is mainly due to the fact that with the increase of YRS, the low activity of YRS does not allow the cementitious materials to endure more depolymerization reaction under the action of alkali exciters. Furthermore, under natural curing, the compressive strength at 3 d is maximum at 40% RM dosage. However, at 28 d, the compressive strength is maximum at the 60% RM dosage. This may be due to the fact that the alkali excitation reaction of YRS requires more water than that of RM, resulting in a later source of strength for cementitious materials with lower water content produced mainly by alkali excitation of RM. Under standard curing, the compressive strength at 28 d is a maximum of (48.8 MPa) at 60% RM dosage, which may be due to the higher pozzolanic activity of RM compared to YRS.

**Figure 5:**
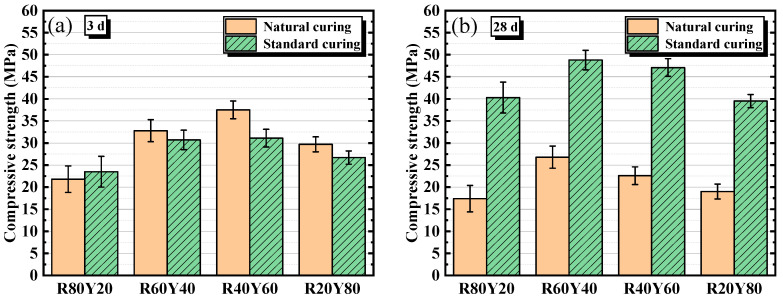
Compressive strength of AGRY cementitious materials at different ages: (**a**) 3 d; (**b**) 28 d.

Figure 5 shows the comparison of compressive strength of AGRY cementitious materials at the same age under different curing conditions. From Figure 5a,b, at age of 3 d, the compressive strength under natural curing is higher than that under standard curing, except for the R80Y20 group. However, at age of 28 d, the compressive strength under standard curing is much higher than that under natural curing. This is mainly due to the fact that at the same alkalinity, the concentration of sodium silicate under standard curing will be diluted by the water from the curing environment at an early age, which results in a lower alkalinity than under natural curing and reduces the alkali-excitation reaction rate. When the curing age is long, most of the water in the cementitious materials under natural curing is consumed which hinders the alkali-excitation reaction. In contrast, the continuous supply of external moisture under standard curing can induce the alkali excitation reaction in the cementitious materials to continue, which results in a gradual increase in strength. Overall, the curing conditions have a great influence on the mechanical properties of AGRY cementitious materials, and the compressive strength under standard curing at 28 d for the same mix ratios can be enhanced by 82~132% compared to that under natural curing.

### 3.3. X-ray Diffraction

**Figure 6:**
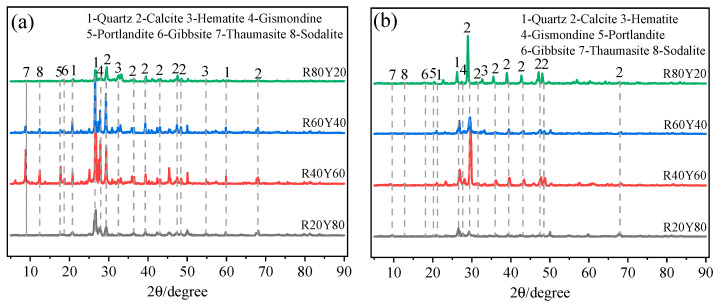
XRD patterns of AGRY cementitious materials at 28 d: (**a**) Natural curing; (**b**) Standard curing.

The XRD patterns of AGRY pastes at 28 d under different curing conditions are shown in Figure 6. Quartz, calcite, hematite, plagioclase zeolite, calcium hydroxide, alumina trihydrate, calcium carbon-sulfosilicate, and galena are found in AGRY paste [69]. Comparison of the plots of the cementitious materials under different curing conditions reveals that the peaks of quartz appear near 27° in both the plots under standard curing and natural curing, and the peaks under standard curing are much lower than those under ordinary curing. This is mainly due to sufficient moisture under standard curing, where the active components in YRS can fully undergo alkali activation reactions, resulting in a reduction of SiO_2_ [22]. Compared to R80Y20 and R20Y80, the characteristic peaks at the location of calcite in R60Y40 and R40Y60 are significantly enhanced, especially at 30°. This indicates that after alkali excitation, the cementitious materials crystallize mainly in the form of calcite [70], which is the main reason for the increase in strength. The reaction of Ca^2+^, Si^4+^, and Mg^2+^ in the cementitious materials with OH^−^ in the alkali exciter is shown in Equations (3)–(5) [69].

Ca^2+^ + CO_2_ + 2OH^−^ = CaCO_3_ (3)

Ca^2+^ + 2OH^−^ = CaO + H_2_O (4)

2CaO + Mg^2+^ + Si^4+^ = 2CaO·MgO·2SiO_2_ (5)

### 3.4. Scanning Electron Microscope

**Figure 7:**
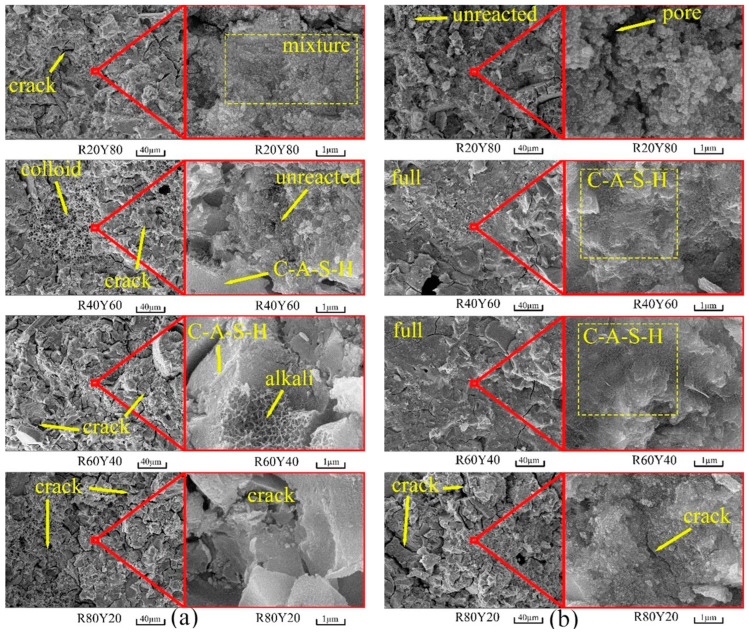
SEM images of AGRY pastes at 28 d: (**a**) natural curing; (**b**) standard curing.

The microscopic morphology of AGRY paste at 28 d is shown in Figure 7. It can be seen that under natural curing (Figure 7a), the microstructure of the AGRY paste is loose and has more honeycomb pores and cracks. This is mainly due to the dry air environment under natural curing, which allows the alkali excitation reaction to be greatly inhibited due to lack of moisture. Also, the AGRY pastes that have undergone the alkali excitation reaction are prone to water loss, resulting in drying cracks. Cracks may lead to a reduction in the internal structural compactness of the AGRY paste, thus affecting the strength, which is consistent with the change in the compressive strength of the cementitious materials in Section 3.2.

Under standard curing (Figure 7b), the microstructure of the AGRY paste is denser, and honeycomb pores and cracks are less frequent. The internal structure of the AGRY paste gradually densifies as the RM dosage increases from 20% to 60%. A large number of gels are produced in the samples of R40Y60 and R60Y40. This is mainly due to the increase in the content of the active ingredient due to the addition of RM in the AGRY paste. However, when the RM dosage is increased from 60% to 80%, more cracks are produced in the AGRY paste. This is primarily a swelling phenomenon due to excessive alkali excitation products formed within the material. When the expansion stress is greater than the stress that the paste can withstand, minor cracks will appear within the paste and result in a reduction in the strength of the AGRY paste.

**Figure 8:**
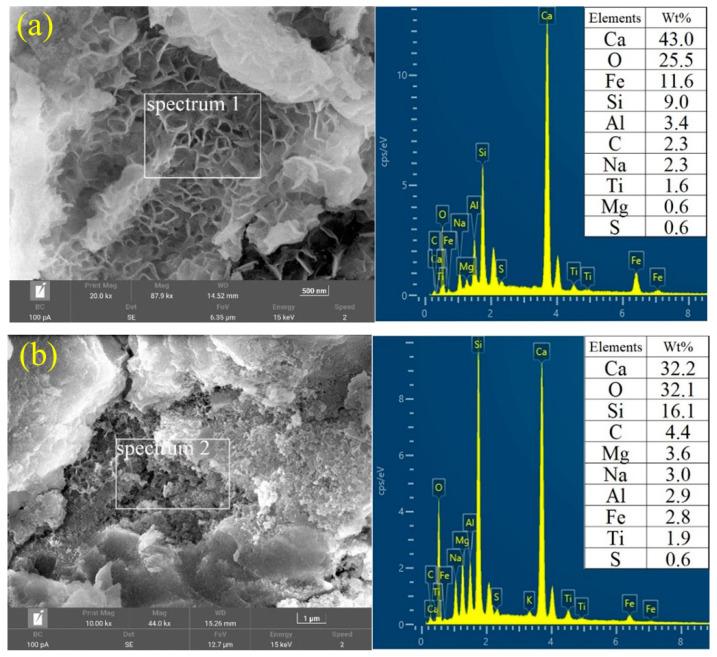
SEM and EDS images of AGRY pastes at 28 d: (**a**) R60Y40-N; (**b**) R60Y40-S.

Figure 8a, b shows the results of EDS face-scanning performed on the microstructures of R60Y40-N and R60Y40-S, respectively. The face-scanning range of EDS is selected for the gel-like product, and a comparison of the elements in the range with the (N, C)-A-S-H gel reveals that they have similar compositions [22]. Meanwhile, the Si content is higher under standard curing than under natural curing, which is directly related to the presence of Si in the C-A-S-H gels. The high Si content of the R60Y40-S group may be due to the fact that the gel products do not exist independently but are dependent on the surface of the high Si content base material YRS. This phenomenon is not evident in the natural curing group, which is due to the fact that the standard curing is more favorable for the less active YRS to participate in the alkali excitation reaction. Further, more O elements are revealed in the paste under standard curing compared to under natural curing. Compared to the raw material, the (N, C)-A-S-H gel contains more O elements. As more gel is generated in the hydration products, the O element is significantly higher. As a result, the products generated by hydration under standard curing contain a higher amount of element O. In addition, the polycondensation process during the alkali-excited reactions generates a large number of oxides [70], which suggests that the alkali-excited reactions with high O content are more intense under standard curing than under natural curing.

Unlike previous studies [22], the elemental Ca content in the (N,C)-A-S-H gels in this study is high. This is mainly due to the high Ca content in RM and GGBS, the raw materials used in this study, and the presence of Ca^2+^ leads to the degradation of most of the N-A-S-H gels, which promotes the generation of C-A-S-H gels [71]. In this ground polymerization reaction, Na_2_O, CaO, Al_2_O_3_, SiO_2_, and H_2_O are involved in the reaction, i.e., high concentrations of Na, Ca, Al, and Si are released at the initial stage of mixing. Thus, Na^+^ and Ca^2+^ act as charge balancers in the structure of SiO_4_ and AlO_4_ and ultimately produce the (N, C)-A-S-H gels.

### 3.5. Mercury Intrusion Porosimetry

**Figure 9:**
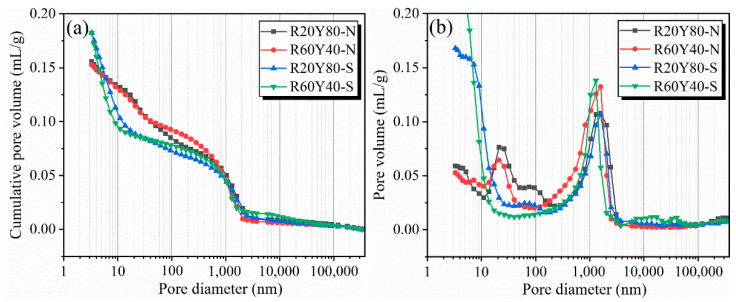
Pore structure distribution of AGRY pastes at 28 d: (**a**) cumulative pore volume; (**b**) pore volume distribution.

The pore structure of AGRY pastes at 28 d under different curing conditions is shown in Figure 9. From Figure 9a, it is observed that the pore cumulative distribution of AGRY paste under different curing conditions shows significant differences, but the pore cumulative distribution under the same curing conditions shows smaller differences. From Figure 9b, it can be seen more clearly that the pore volume of R20Y80-S and R60Y40-S with a diameter of less than 10 nm under standard curing is much higher than that of R20Y80-N and R60Y40-N under natural curing. Meanwhile, the observation of the peaks at pore sizes of 200–3000 nm in Figure 9b reveals that the peaks of R60Y40 are higher than those of R20Y80 under the same curing conditions. According to the relevant research, the holes with pore size less than 20 nm are harmless holes, the holes with pore size 20–50 nm are less harmful holes, the holes with pore size 50–200 nm are harmful holes, and the holes with pore size > 200 nm are multi-hazardous holes [72]. This suggests that an increase in YRS reduces the number of multi-hazardous holes and thus improves the strength of cementitious materials. In addition, the presence of two peaks at 15~30 nm and 500~3000 nm is observed for the pore volume of different pore sizes in the R20Y80 and R60Y40 groups under natural curing.

**Figure 10:**
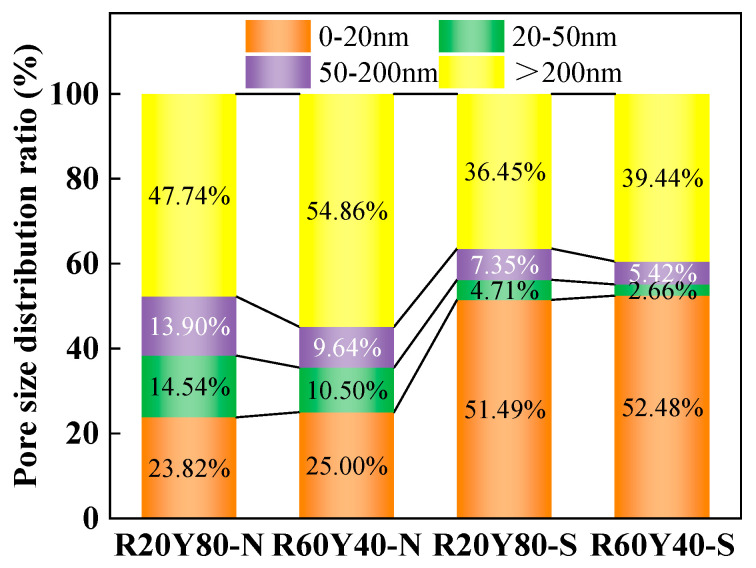
Pore size distribution ratio of AGRY pastes.

The pore size distribution is processed for the pore results and shown in Figure 10 and Table 3. It can be seen that there are more harmless pores below 20 nm under standard curing compared to natural curing. In the case of R20Y80, for example, the porosity below 20 nm under natural curing accounts for 23.82%, while the porosity under standard curing is 51.49%, so the enhancement of the densification of the paste by standard curing is very significant compared to natural curing. In addition, there is a significant increase in the number of multi-hazardous holes above 200 nm under natural curing compared to that under standard curing. In the case of R60Y40, for example, the porosity above 200 nm is 54.86% under natural curing, while it is 39.44% under standard curing. This is mainly due to the fact that the hydration of the paste under standard curing is more sufficient to produce more hydration products with cementitious ability, which results in a denser structure.

### 3.6. Fourier Transform Infrared Spectroscopy

**Figure 11:**
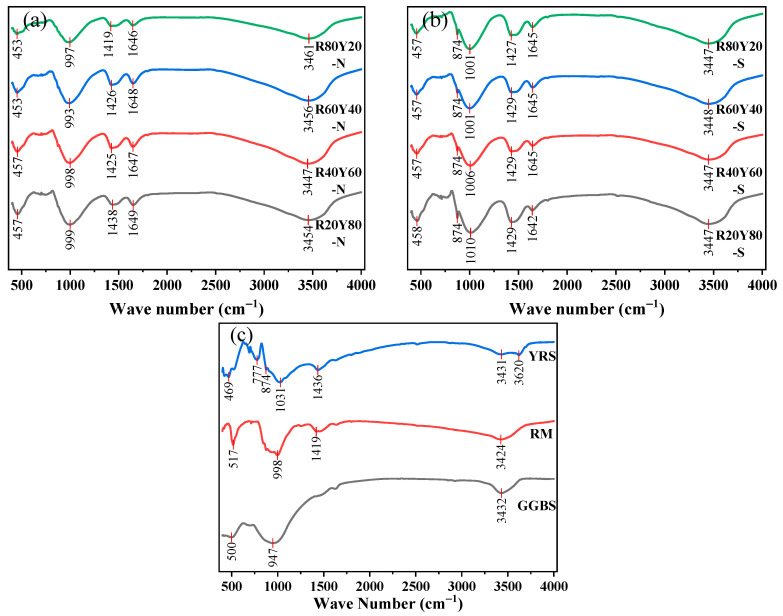
FTIR spectra of AGRY cementitious materials: (**a**) natural curing; (**b**) standard conservation; (**c**) raw materials.

Figure 11 shows the FTIR spectra of YRS, RM, and GGBS, and AGRY pastes under different curing conditions. Significant characteristic peaks are observed at 453~457 cm^−1^ for all samples, which are caused by the bending vibrations of Si-O in the SiO_4_ tetrahedra. In this band, the characteristic peaks of the AGRY pastes are shifted in the direction of lower wave numbers compared to the raw materials, and the reason for this shift comes from changes in crystallinity due to structural orders [73]. The increase in structural order implies an increase in the internal ordering of the material, which may lead to a redshift of the Si-O vibrational modes, i.e., towards lower wave numbers, suggesting that the geopolymers prepared with different coordination ratios produce slightly different lattice morphologies during condensation. Further, it is observed that all the samples have characteristic peaks in the ranges of 997~1010 cm^−1^ and 947~1031 cm^−1^ and the peaks will be shifted towards higher wave numbers under standard curing, corresponding to asymmetric stretching vibrations of Si-O-Si and Si-O-Al. This suggests that the formation of polymers in the paste may be related to the C-A-S-H structure [74]. Thus, the characteristic peaks of Si-O-Si or Si-O-Al can be used to characterize the formation of polymer gels. Stretching bands are also observed within 1419 to 1438 cm^−1^ and 1427 to 1429 cm^−1^ in the natural curing group. This is caused by the stretching vibrations of O-C-O in the carbonate group, which can be defined as the characteristic band of calcite. In addition, distinct characteristic peaks are also observed at 1642~1649 cm^−1^ versus 3447~3461 cm^−1^, which are caused by O-H vibrations and can be attributed to water molecules adsorbed on the surface of the gel or water molecules inside the raw materials. However, there is no significant characteristic peak for the cementitious material containing 40% RM with high strength as compared to other groups.

### 3.7. Thermogravimetric Analysis

**Figure 12:**
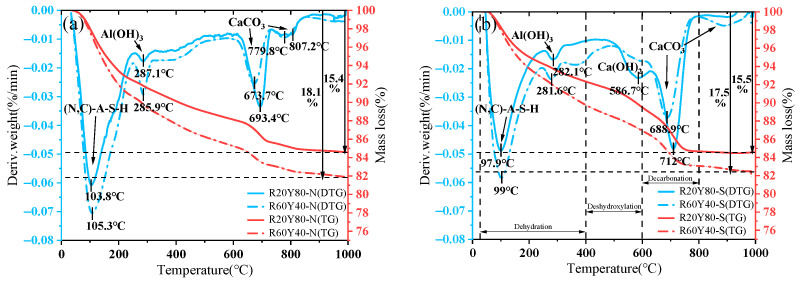
TG and DTG of AGRY pastes: (**a**) natural curing; (**b**) standard curing.

The TG results of AGRY pastes under different curing conditions are shown in Figure 12. Studies have shown that cementitious materials in the continuous heating and warming process of geopolymers will produce a series of physical and chemical reactions, its weight will gradually decrease, and the reasons for the loss of mass at different temperatures are not the same [75]. In general, there are three main stages of mass loss in cementitious materials during continuous warming [76]: (1) the loss of free and bound water in the range of 25~400 °C; (2) 400~600 °C corresponds to the dehydroxylation of Ca(OH)_2_; and (3) the process of decarbonylation of CaCO_3_ is predominant between 600 °C and 800 °C.

From the TG curves, it can be seen that the mass loss rates of the four groups of AGRY pastes are 15.4% (R20Y80-N), 18.1% (R60Y40-N), 15.5% (R20Y80-S), and 17.5% (R60Y40-S) after ramping up the temperature to 1000 °C, respectively. Therefore, the increase in RM increases the thermogravimetric loss of the AGRY pastes under the same curing conditions. The mass losses of R20Y80-N, R60Y40-N, R20Y80-S, and R60Y40-S at 600~800 °C are 3.0%, 2.7%, 4.8%, and 3.9%, respectively. This temperature interval corresponds to the decarbonization of CaCO_3_. It can be seen that the increase in YRS dosage with wetter standard curing leads to an increase in the mass loss of CaCO_3_, which is a result that is consistent with the XRD results.

From the DTG curves, it can be seen that a significant heat absorption peak is observed in all four AGRY pastes at about 97.9~105.3 °C. The mass loss in this temperature interval is mainly related to the evaporation of gel products (e.g., C-S-H, C-A-S-H, and N-A-S-H gels) and free water. It is also observed that the peaks of mass loss between 97.9 °C and 105.3 °C are enhanced with increasing RM dosage, and the peaks are shifted to higher temperatures. This is mainly due to the fact that higher RM dosage is more favorable for gel formation, making the gel structure more complex. The complex gel structure has a higher ability to immobilize water molecules, thereby increasing the temperature required to evaporate water. Further, a smaller peak is observed near 281.6~287.1 °C, corresponding to the decomposition of Al(OH)_3_. In addition, a peak of dehydroxylation of Ca(OH)_2_ is observed near 586.7 °C and a distinct peak of decarbonylation of CaCO_3_ is observed from 673.7 °C to 712 °C under standard curing.

### 3.8. Cost, Carbon Emissions, and Multi-Criteria Performance Assessment Analysis

The environmental and economic impact statistics are shown in Table 4. According to the market inquiry, the four-quarter average price of GGBS in 2023 was CNY 44/t. The sodium silicate of 2.3 M used in this study had a four-quarter average price of CNY 262/t in 2022. It is calculated that the cost of raw materials consumed for the preparation of each cubic meter of AGRY paste is about CNY 79/m^3^, which is lower than the market price of CNY 87/m^3^ of ordinary silicate cement (P-II 52.5R) paste. In this study, the use of new cementitious materials instead of cement as raw materials for the development of concrete is one of the important factors in reducing carbon emissions. Therefore, based on the data from previous studies, the carbon emissions of industrial sodium silicate, silicate cement, and GGBS are 1.514 t CO_2_/t, 0.82 t CO_2_/t, and 0.143 t CO_2_/t, respectively [70,77,78]. The CO_2_ emissions of AGRY paste and ordinary silicate cement paste are then calculated separately. Calculations show that the production process generates about 0.41 t CO_2_ per 1 m^3^ of the material required for the production of AGRY paste and about 0.95 t CO_2_ per 1 m^3^ of the cement required for the production of cement paste.

The compressive strength of AGRY cementitious materials in this study is close to that of the cement (P-II 52.5R) paste, so its cost and carbon emissions are compared. The cost of AGRY cementitious materials is about 10% lower than that of cementitious materials, and the carbon emission of AGRY cementitious materials is only 43% of that of cementitious materials for the same volume.

**Figure 13:**
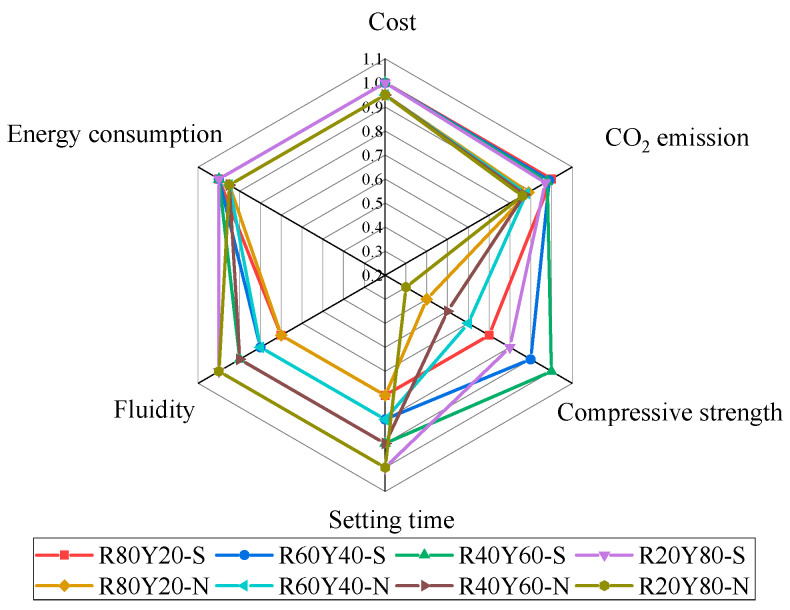
Performance evaluation of AGRY cementitious materials.

In this study, the six factors of cost, carbon emission, mechanical properties, setting time, fluidity, and energy consumption of cementitious materials are summarized and integrated in the form of radar charts to evaluate and analyze them. From Figure 13, it can be seen that the mechanical properties of AGRY pastes are directly proportional to the carbon emissions but have little relationship with the setting time and fluidity. Meanwhile, the fluidity of cementitious materials is directly proportional to the setting time. The change in carbon emissions of the cementitious materials in this experiment is not significant, and the difference mainly comes from the elevated emission from the standard curing method and the CO_2_ emission from the RM hydration process in the more reactive mixing ratios. It can also be observed that the cost of the cementitious materials and the carbon emissions have a small effect on the strength of cementitious materials, which means that some of the cost and carbon emissions can be sacrificed in exchange for a significant increase in the strength of cementitious materials.

## 4. Conclusions

In this work, utilizing the basic principles of alkali-excited materials, the new AGRY cementitious materials with excellent mechanical strength, low cost, and low carbon emission were prepared by using two synergistic matrix materials, RM and YRS. Based on the macro and micro results obtained from the study, the following conclusions can be drawn:

(1) The addition of YRS can improve the fluidity and setting time of AGRY cementitious materials. Compared with the cementitious material with 80% RM and 20% YRS, the fluidity of the cementitious material with 20% RM and 80% YRS is improved from 62 mm to 220 mm, and the final setting time is extended from 3 min to 23 min.

(2) Natural curing improves the early compressive strength of AGRY cementitious materials, while the compressive strength decreases gradually with the increase of age. Standard curing improves the later compressive strength of AGRY cementitious materials. The compressive strength at 28 d under standard curing for the same mix ratios is 82–132% higher than that under natural curing. Under standard curing, when the mass ratio of RM to YRS is 60: 40, the compressive strength at 28 d can reach 48.8 MPa and is close to that of the ordinary silicate cement (P-II 52.5R).

(3) The sufficient moisture under standard curing can make the AGRY cementitious materials at 28 d obtain a more adequate alkali excitation reaction and generate more hydration products than that under natural curing. A higher RM dosage is more favorable for the formation of (N, C)-A-S-H gels and makes the gel structure more complex.

(4) Under natural curing, the microstructure of the AGRY cementitious materials is loose and has more honeycomb pores and cracks. Under standard curing, the microstructure of the AGRY cementitious materials is denser, and the honeycomb pores and cracks are less frequent. The AGRY cementitious material under standard curing at 28 d has more harmless pores below 20 nm compared to that under natural curing.

(5) The solid wastes, RM, and YRS can be utilized resourcefully, and Al in RM, and Si and Na in YRS can well make up for the sodium silicate required for the formation of (N, C)-A-S-H and reduce the amount of high-cost alkali exciters. The cost of AGRY cementitious materials in this study is about 90% of the cost of cementitious materials, and the carbon emission of AGRY cementitious materials is about 43% of that of the ordinary silicate cement.

The prepared AGRY cementitious materials offer great possibilities for replacing cementitious materials in concrete. However, there are still some problems in the process of this study, the limitations of this study and possible future studies are as follows:

(1) The setting time of AGRY cementitious materials prepared in this study is short, which is not enough to meet the requirements of practical engineering. Therefore, it is still necessary to further explore the method of prolonging the setting time of AGRY cementitious materials.

(2) Although low-carbon and high-strength AGRY cementitious materials are prepared in this study, their durability has not been effectively demonstrated, so there are still some problems in the practical application of AGRY cementitious materials in buildings.

## Figures and Tables

**Table 1:** Chemical compositions of GGBS, RM, and YRS.

Oxides (wt%)	CaO	SiO_2_	Fe_2_O_3_	Al_2_O_3_	TiO_2_	Na_2_O	SO_3_	K_2_O	MgO	Other
GGBS	49.42	25.57	0.31	13.58	2.15	0.44	2.36	0.33	5.32	0.52
RM	55.54	18.30	8.31	6.48	5.08	3.12	0.89	0.84	0.81	0.63
YRS	8.90	65.67	4.76	10.87	0.85	2.79	0.10	2.77	2.62	0.67

**Table 2:** Mix proportions of the materials required per liter of AGRY cementitious material (g/L).

Specimens	GGBS (g)	RM (g)	YRS (g)	Water (g)	W/(RM + GGBS + YRS)	Alkali Exciter (g)	Water-Reducing Agent (g)
R80Y20-N R80Y20-S	360	672	168	480	0.4	240	6
R60Y40-N R60Y40-S	360	504	336	480	0.4	240	6
R40Y60-N R40Y40-S	360	336	504	480	0.4	240	6
R20Y80-N R20Y80-S	360	168	672	480	0.4	240	6

N is natural curing and S is standard curing.

**Table 3:** MIP results of AGRY pastes.

Specimens	Porosity (%)	0–20 nm (%)	20–50 nm (%)	50–200 nm (%)	>200 nm (%)
R20Y80-N	27.28	23.82	14.54	13.90	47.74
R60Y40-N	26.57	25.00	10.50	9.64	54.85
R20Y80-S	28.62	51.49	4.71	7.35	36.45
R60Y40-S	27.80	52.48	2.66	5.42	39.44

**Table 4:** Estimates of environmental and economic effects.

Items	Energy Usage (GJ/t)	Electricity Usage (GJ/t)	Greenhouse Gas Emission (CO_2-eq_/t)	Approx. Cost (Market Price)
RM	-	-	-	0
YRS	-	-	-	0
GGBS	-	-	0.143 [70]	CNY 44/t
Sodium silicate	4.3	0.30	1.514 [78]	CNY 262/t
Cement	5.0	0.54	0.82 [77]	CNY 87/m^3^
R60Y40-S	1.4	1.10	0.41	CNY 79/m^3^

## Data Availability

The raw data supporting the conclusions of this article will be made available by the authors on request.
